# *The last among the first:* Christian Fredrik Borchgrevink (1924–2024) in Memoriam

**DOI:** 10.1080/02813432.2024.2442845

**Published:** 2024-12-24

**Authors:** Jørund Straand, Per Olav Hjortdahl, Dag Bruusgaard

**Affiliations:** Department of General Practice, Institute of Health and Society, University of Oslo, Norway


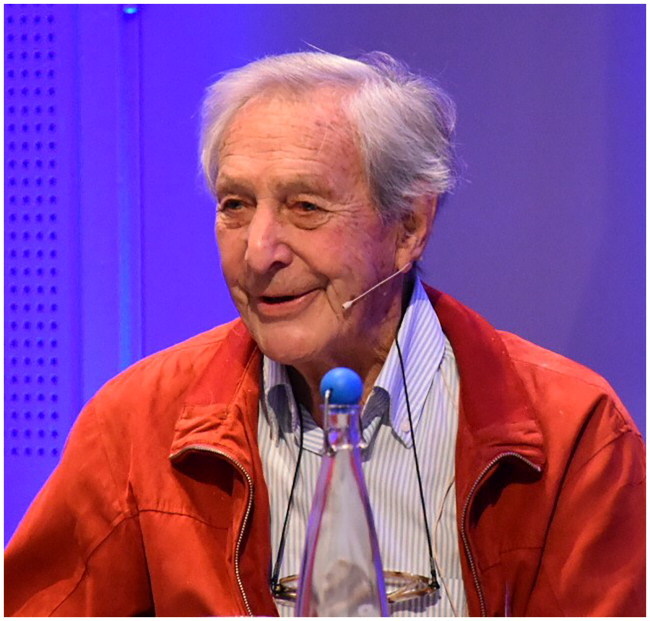


Christian Fredrik Borchgrevink, the first Nordic professor of general practice and former Editor-in-Chief of the *Scandinavian Journal of Primary Health Care*, passed away on October 13, 2024, six weeks after he had celebrated his 100^th^ birthday.

After graduating from medical school in Oslo in 1951, Christian Borchgrevink spent a year as a district physician on Norway’s west coast. He later remarked that it was during this time that he truly adopted the mindset of a general practitioner. However, his early career path took different turns. He worked abroad in Poland and Indonesia before pursuing internal medicine at the National Hospital in Oslo. In 1961, he defended his doctoral thesis on anticoagulation and heart disease.

In late 1968, the University of Oslo established The Institute of General Practice funded by the Norwegian Medical Association. Borchgrevink became the institute’s first professor. This made him the world’s fourth professor of general practice, following Richard Scott (Edinburgh, 1963), Jan van Es (Utrecht, 1966), and Ian McWhinney (Ontario, 1968) [1]. It is noteworthy that, in 1968, he was also offered a professorship in internal medicine at the university, which he declined.

As a university professor and chair of the department, he inspired countless medical students to pursue careers in general practice. He was the driving force behind the founding of the Norwegian Society for General Practice (NSAM) in 1983, which later became the Norwegian College of General Practice (NFA). From 1989 to 1996, Borchgrevink served as Editor-in-Chief of the Scandinavian Journal of Primary Health Care, with Karin Dolven (1931–2023) as his Editorial Secretary. Professor Borchgrevink was also a prolific writer on a wide range of medical topics.

Professor Borchgrevink was a member of the Medical Association’s central board from 1971 to 1977, serving as vice president during the two last years. He was also a key figure in the National Association against AIDS and a cofounder of Norwegian Doctors Against Nuclear Weapons. Under his leadership, The Institute of General Practice at the University of Oslo supported the development of primary health care services in northern Portugal following the Carnation Revolution of 1974.

After 25 years at the University of Oslo, Borchgrevink retired in 1994. But he continued to supervise GP researchers and even maintained a small clinical practice until the age of 96. For his contributions, he was honored with knighthood in the prestigious Norwegian Order of St. Olav.

We remember Christian as an enthusiastic, tolerant, playful friend and colleague, with a well-developed sense of humor and a razor-sharp intellect. During his centenary celebration, he heard firsthand how deeply his work had impacted so many.

CFB was the last surviving member of the first four professors of general practice in the world. His legacy in shaping and elevating the profession of general practice both nationally and internationally is immeasurable.
